# The Use of Medicinal Plants in Maceió, Northeastern Brazil: An Ethnobotanical Survey

**DOI:** 10.3390/medicines7020007

**Published:** 2020-01-21

**Authors:** Thycia Maria Gama Cerqueira, Ana Carolina de Carvalho Correia, Rafael Vital dos Santos, Rosângela P. Lyra Lemos, Sâmia Andrícia Souza da Silva, Emiliano Barreto

**Affiliations:** 1 Laboratório de Biologia Celular, Maceió 57072-970, AL, Brazil; thycia10@hotmail.com (T.M.G.C.);; 2 Garanhuns College of Science, Education and Technology, University of Pernambuco, Garanhuns 55294-902, Brazil; anacarolinacc@yahoo.com.br; 3 Instituto do Meio Ambiente do Estado de Alagoas, Maceió 57017-320, AL, Brazil; rosalyralemos@gmail.com; 4 Escola de Enfermagem e Farmácia, Universidade Federal de Alagoas, Maceió 57072-970, AL, Brazil; samanndri@yahoo.com.br

**Keywords:** ethnobotanical study, medicinal plants, northeast of Brazil

## Abstract

**Background:** The purpose of this study was to record and analyze the knowledge of medicinal plant use in the community in urban areas of Maceió city, Brazil. **Methods:** A total of 113 patients from the basic healthcare unit were assessed. **Results:** Approximately 95% of the interviewed stated that the plants were used for medicinal purposes. The majority of respondents were women (94.7%) who were between 51-60 years of age. Forty-eight plant species belonging to 28 families were cited as useful for medicinal purposes. The main families encountered were Lamiaceae (16.6%), Asteraceae (8.3%), Myrtaceae (6.2%), Fabaceae (6.2%), Annonaceae (4.1%), Laureaceae (4.1%), Rutaceae (4.1%), and Zingiberaceae (4.1%). These plants were used to treat a wide range of disturbances, including gastrointestinal, respiratory, and cardiovascular diseases. The majority of the respondents used decoctions of leaves that were cultivated in house (58.4%) to make their herbal preparations. The respondents revealed that medicinal plant preparations were safe and unaware of that are risks associated with their use. **Conclusions:** Medicinal plants still play an important role in the medical practices of the urban population from Maceió, Brazil. Our results highlight the importance of these plants for local people and indicate the need for further scientific investigations to validate their use as a complementary therapy for disease control.

## 1. Introduction

Brazil has great biodiversity, and local people who have direct access to nature and the products of biodiversity have accumulated a wealth of medicinal knowledge of plants. In Northeast Brazil, the Caatinga, and the Tropical Atlantic Rainforest represent the main biome of this region [1]. In this region, the use of plants to medicinal ends is a very common practice that plays an important role as a therapeutic alternative [2]. There has been increasing interest in acquiring knowledge of the medicinal plants in these areas due to publications that have described that the rich flora of this region have many therapeutic uses [3,4,5,6]. Alternative therapies have been increasingly accepted and they are used in many countries in the world, including European countries, North America, and Australia [7]. In Brazil, these therapies have been integrated into health care due to their promotional, preventive, curative, and rehabilitative effects in many acute and chronic diseases [8].

In recent years, there has been an increase in the number of patients that visit primary healthcare units, which leads to an increase in the demand for hospitalization. In addition, the financial and/or geographical accessibility of some populations to allopathic drugs is limited. These trends lead to the increasing use of traditional remedies by certain populations as the first available approach. Thus, the conservation of the ethnobotanical knowledge that is part of living cultural knowledge and practice between society and the environment is necessary for the population’s health [9]. Even in urban populations, traditional medical knowledge regarding medicinal plants and their use is vital for the preservation of cultural traditions and biodiversity.

Traditionally, this knowledge is passed on through generations by oral tradition. Thus, there is an urgent need for ethnobotanical surveys to document the wealth of knowledge of folk medicine before it is lost by modernization. Furthermore, these ethnobotanical approaches of traditional medicine guarantee the preservation of local cultural traditions and biodiversity.

Previous reports revealed that studies on medicinal plants in Brazil are found in other states, such as Paraná [10], Mato Grosso [11], Rio de Janeiro [12], and Pará [13]. In Alagoas, there is a dearth of information and documentation regarding the knowledge that is involved in traditional medicine. In addition, data that assess the use and management of medicinal plant species in basic healthcare units from in Maceió and specifically from the present study area are lacking. Therefore, the purpose of this survey was to identify and document the local native plants that are used by the people living in urban areas in Maceió city, Brazil.

## 2. Materials and Methods

### 2.1. Study Area

The present study was conducted in a basic healthcare unit in São José Canaã, which is located between the districts of Santo Amaro and Canaã, in the municipality of Maceió, Alagoas, Brazil. This basic healthcare unity was selected due to its geographic position in the central area of the city of Maceió and because it provides healthcare service to the entire population of neighboring areas. The general area of coverage of the basic healthcare unit in São José Canaã is 1.5 km^2^ and it extends from about 09°28′14″ to 09°42′42″ S and from 35°33′29″ to 35°47′38″ W.

### 2.2. Population Sample and Data Collection

**Figure 1:**
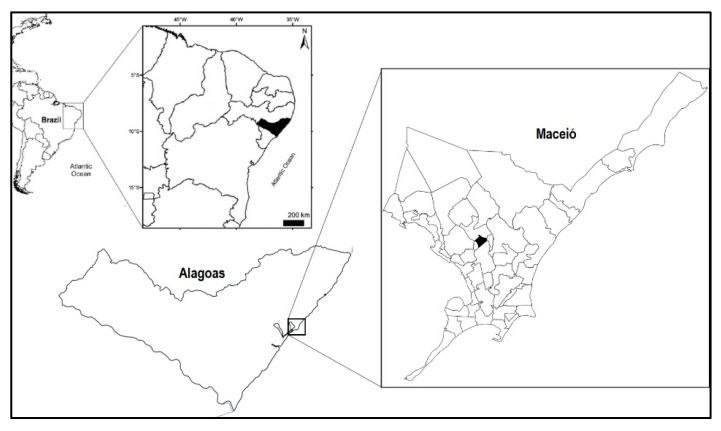
Geographical location of the study area.

The basic healthcare unit of São José Canaã (Figure 1) had 9479 registered users being the target population of study users with ages that were equal to or over 40 (2017 users). The following parameters were used to determine the number of users that needed to be evaluated for sufficient power: 9.8% acceptable error, a confidence interval of 95%, and an expected frequency of 80% for the use of medicinal plants, according to a current estimate by the World Health Organization [14]. The sample size calculation demonstrated the need for a minimum sample of 118 users. The field data were collected from June 2018 to September 2019.

A questionnaire with structured and semi-structured questions was used to interview the users. The informants were asked about the vernacular names, the part used, the methods of preparation, the administration routes, and the medicinal indications of any plants that were used for medicinal purposes. The interviews and discussions were conducted while using a local dialect to allow for easy communication with the participants. The Research Ethics Committee of the Federal University of Alagoas reviewed and approved all of the research procedures (Protocol no. 021671/2017-35), and all of the volunteers signed a Terms of Informed Consent Form at the start of the research. The approval date (May 25, 2017).

### 2.3. Identification of the Botanic Material

The species that were described by the respondents were collected in gardens in close proximity to or in their respective homes. Voucher specimens were preserved in the herbarium of the Instituto do Meio Ambiente do Estado de Alagoas. The specimens were identified for specialists from the herbarium of the Instituto do Meio Ambiente do Estado de Alagoas and confirmed by means of The Plant List site [15].

### 2.4. Data Analysis

Ethno-medicinal documentation needs to validate data through numero-indices while using quantitative analysis. Among the quantitative approaches informant consensus factor (ICF) and the use-value index (UV) aim to quantitatively describe the variables and analyze the observed patterns in the study [16]. Here, the Informant Consensus Factor (ICF) was employed to determine the homogeneity in the information that was given by the informants. All of the citations were placed into 10 categories (nervous system disorders, fever, gastrointestinal disorders, urogenital disorders, pain, healing and inflammation, diabetes, respiratory diseases, cardiovascular diseases, and cancer). The ICF was calculated as the number of use citations in each category (Nur), minus the number of species used (Nt), divided by the number of use citations in each category, minus one:

$$\mathrm{ICF}=\frac{\left(\mathrm{Nur}-\mathrm{Nt}\right)}{\left(\mathrm{Nur}-1\right)}$$

ICF values range from 0.00 to 1.00. High ICF values are obtained when only one or a few plant species have been reported to be used by a high proportion of informants to treat a particular category, whereas low ICF values indicate that informants disagree over which plant to use [16]. In addition, the use-value (UV) index was employed to calculate the citation of the plants after the interviews, and it was calculated, as follows:

$$\mathrm{UV}=\frac{U}{N}$$

where U is the sum of the total number of use citations by all informants for a given species divided by the total number of informants (N). This method evaluates the relative importance of each medicinal species based on its relative use among the informants [16].

## 3. Results and Discussion

The discovery and disclosure of medicinal flora properties through the knowledge of various populations is an important tool in preserving the cultural richness of a people. In the present study, information regarding the medicinal properties of 48 species was reported. The most important finding was the demonstration, for the first time, that these traditional medicinal plants still play an important role in the medical practices of individuals who live in urban areas of Maceió, Alagoas, Brazil.

In our study, we interviewed 118 informants living in an urban area of Maceió for ethnomedicinal investigation of the plants that were used for medicinal purposes. The demographic characteristics of the respondents were determined and recorded through face-to-face interviews. We found that 94.2% (113) of the total group of respondents were users of medicinal plants for therapeutic purposes. Of the participants who took part in the questionnaire, the large majority of users were women (94.7%) that were between 51 and 60 years of age (Table 1). 

According to Table 1, the vast majority of respondents consisted of illiterate (no schooling) individuals and individuals with a maximum educational status of the primary level. Among the respondents, 58.0% had a family income between 300–600 dollars per month. In addition, another percentage of the respondents (34.5%) lived on less than 300 dollars per month. These observations were in line with the results of a study conducted by Delgoda, et al. [17], which has shown a strong association between the use of medicinal herbs and low education and family income. In addition, our study revealed that most of the users (77.9%) used 1–3 different types of medicinal plants to treat their illnesses and their family was the main source of the information that they knew about the use of these plants, according to the statements that were made by raizeiros (38.5%) (Table 1).

All of the observations that were made in this study were in line with those of other populations in the Brazilian Northeast, as well as in other Brazilian regions, revealing a characteristic of the Brazilian culture of the use of medicinal plants [6,18]. It should also be mentioned that, the present study was performed with only patients assisted under a Basic Healthcare Unit from Brazilian public health system (SUS), although other authors have analyzed the profile of use of medicinal plants in Maceió city [19]. The analysis of this population is particularly important for the proper care to the type of individual that seeks the public health system.

In this study, we needed to standardize the information that was obtained by the questionnaires by using the Latin name of the plants that were described by the population to minimize the errors of indication due to variations in the common names of plants. We can illustrate this case by using the species *Chenopodium ambrosioides* L., which is known by the population as “mastruz” [20]. However, in the southern and southeastern regions of Brazil, it is known as "erva-de-Santa-Maria" [21]. It is worth mentioning that these variations in the popular names of plants mainly occur because of ethnographic reasons in Brazil. In addition, the species can presents many names, as *Plectranthus barbatus* that has commonly been referred to as *Plectranthus forskohlii* Briq, *Plectranthus forskalaei* Willd., *Plectranthus kilimandschari* (Gürke) H.L. Maass., and *Plectranthus grandis* (Cramer) R.H. Willemse [22]. Thus, the botanical identification of plants is a very important issue, especially for understanding ethnomedicinal use by population.

We found that 48 distinct plants were used for curative purposes in this study. The data that were collected from this ethnomedicinal survey were analyzed to evaluate the relative importance of each plant to the population. The species were distributed in 28 families and 45 genera (Table 2). Lamiaceae (16.6%) and Asteraceae (8.3%) were the families with the largest number of species used, which were followed by the families Myrtaceae (6.2%), Fabaceae (6.2%), Annonaceae (4.1%), Laureaceae (4.1%), Rutaceae (4.1%), Zingiberaceae (4.1%), and 22 other families (2% each). Other studies have reported the importance of the Lamiaceae and Asteraceae families, which have been the most represented plants in investigations of medicinal plants in the world [16,19,21].

Of the 48 species included herein, most of the species (70.8%) have several medicinal uses and different modes of preparation. The species with the highest number of therapeutic indications was *Chenopodium ambrosioides* (8), *Lippia alba* (8), and *Mentha piperita* (8). Fourteen species were cited as useful for only one medicinal indication (Table 2). In most of the cases, the leaves were the most frequently used plant parts for the medicinal preparations (90.2%). Of all the medicinal preparations, infusions and decoctions that were orally used (92.9%) were the most preferred methods.

According to the calculation that was made for the use value (UV), *Lippia alba* (0.81), *Plectranthus barbatus* (0.71), and *Cymbopogon citratus* (0.62) had the highest use value. These plants with high UVs were confirmed as being important in the practice of local traditional medicine due to their high degrees of citation by the respondents (Table 2).

In the Brazilian Northeast, the leaves of *Plectranthus barbatus* are utilized as a digestive aid and to help renal and hepatic problems and intestinal pains [2]. The medicinal indications that were obtained in this study were consistent with these findings.

de Almeida Cde, et al. [23] have also shown the importance of medicinal plants in the Brazilian Northeast. In the Almeida study, *Lippia alba* also had a higher degree of citation and, while considering the number of mentions, values of 0.478 and 0.411 were used in the communities of Bom Sucesso and Cachoeira, respectively. In addition, an extensive review of the *L. alba* species in Brazil showed a high incidence of its use for the same indications that were observed in the population in the present study [23].

*Cymbopogon citratus* is one of the most commonly used plants in Brazilian folk medicine for the treatment of nervous and gastrointestinal disturbances [24]^.^ The tea from the leaves of this plant is popularly used in Brazil as a spasmolytic, analgesic, anti-inflammatory, antipyretic, diuretic, and tranquilizer [25]. *C. citratus* had a use value of 0.57 in a study by de Albuquerque and de Oliveira [26], and it was most used by the population in a semi-arid region of the Pernambuco State, NE Brazil, for the treatment of gastrointestinal problems, such as stomachache and diarrhea. Thus, we note that the importance of traditional knowledge and the indications of this species by the population in study were in agreement with the indications of Brazilian traditional medicine. Thus, knowing the use value of a species might indicate its usefulness to a population by considering its pharmacological features. In addition, a pharmacological activity study with the plants that are being used by local people is quite promising for discovering new therapeutic agents [27].

In this study, the ailments that were reported by the respondents were grouped into 10 categories that were based on the information gathered from the interviewees. The total number of use citations and the total number of plants mentioned for each category were calculated (Table 3). Thus, we noted that nervous system disorders showed a high value (ICF = 0.89), with the species *Lippia alba* (leaf) and *Cymbopogon citratus* (leaf) being the most cited by the respondents. Fever scored the second highest ICF value (0.81), with the species *Eucalyptus tereticornis* (leaf and fruit) and *Sambucus Nigra* (leaf and flows) being the most commonly used to lower the body temperature. Gastrointestinal disorders scored the third highest ICF value (0.75), with the species *Plectranthus barbatus* (leaf), *Lippia alba* (leaf), and *Cymbopogon citratus* (leaf) being the most used to treat these disorders.

Twenty plants with 54 citations were recorded for the treatment of pain. *Mentha piperita* (leaf and flower) and *Cymbopogon citratus* (leaf) were used to treat painful conditions. Thirteen plants with 25 citations were recorded to treat wounds and inflammations (ICF = 0.64), with species *Hyptis pectinata* (leaf), *Schinus terebinthifolius* (leaf), and *Abarema cochliacarpos* (stem-bark, leaves) being the most widely used by the population. Fifteen plants with 21 citations were recorded to treat respiratory diseases (ICF = 0.63), with the species *Mentha piperita* (leaf) and *Coleus amboinicus* (leaf) being the most used in this category.

Comparatively, other diseases, such as diabetes (0.33) and cardiovascular diseases (0.33), presented low scores. Thus, we think that the level of the sharing of the knowledge of the use of medicinal plants amongst the respondents to treat these diseases was relatively lower than that of other diseases because the ICF value portrays the proportion of the uses of plants by various people for a certain category of disease.

After analyzing the scientific literature, we observed that the plants most often cited in our study already had some pharmacological screenings that confirmed the indications for the use of these species by the population. In fact, animal model studies have demonstrated that the *Mentha piperita* species exhibits a relaxant effect on gastrointestinal tissue, analgesic and anesthetic effects in the central and peripheral nervous system, and immunomodulator and chemopreventive actions [28]. Moreover, Viana, Vale, Pinho, and Matos [29] used different animal models for the study of pain and reported the antinociceptive effects of the essential oil that was obtained from *Cymbopogon citratus*. Similarly, Silva, et al. [30] have shown that the essential oil from *Eucalyptus tereticornis* also has analgesic and anti-inflammatory effects. These findings reaffirm the importance of the species from the genus Eucalyptus for Brazilian traditional medicine when used to treat pain and fever.

Similarly, the species *Sambucus nigra* L., *Hyptis pectinata* L. Poits, and *Schinus terebinthifolius*, which were mentioned in this study, have been used by the population to treat respiratory diseases, cancer, and vascular disorders, respectively. It is believed that the mechanisms that are involved in the therapeutic actions of these species are related to the inhibition of pro-inflammatory mediators, such as nitric oxide and PGE_2_ [31,32].

Pharmacological experiments have shown that the hydroethanolic extract of the barks from *Abarema cochliacarpos* (Gomes) Barneby & J.W. Grimes present healing properties and bactericidal activity. These observations are consistent with the descriptions that were obtained in the Brazilian communities of the effects of the extracts and infusions that were obtained from this species for the treatment of infected wounds [33].

Another important aspect of our results was related to the safety of the use of preparations that are homemade from medicinal plants. In our study, most of the interviewees revealed that the preparations that were obtained from medicinal plants were safe and the informants were unaware of the risks that were associated with their use. This result was in agreement with the results of a study by Azaizeh, et al. [34], which revealed that the indiscriminate use of medicinal preparations from plants by the population was due to their lack of knowledge regarding the possible existence of toxicity and even their proven actions. With respect to this matter, we observed that the respondents made use of species that have been reported in the literature as having toxic effects or that were contraindicated for use, such as the species *Ruta graveolens*, which has been described as having abortifacient actions [35].

## 4. Conclusions

The current study documented, for the first time, the traditional knowledge of medicinal plants of patients assisted in a basic health care unit in Maceió-AL, Brazil. For the different plants that were reported to be used for medicinal purpose, there have been scientific studies reporting the pharmacological properties of their active components, which revealed a good association between ethnomedicinal uses and the therapeutic actions of the plant species. This documentation of the traditional uses of medicinal plants in Maceio that can contribute to the current knowledge can improve the relationship between traditional medicine and the pharmaceutical industry. However, further investigation of the use of products that were derived from plants and their toxicological aspects should be conducted to ensure the safety and health of the population.

## Figures and Tables

**Table 1:** Socio-economic characteristic of the respondents.

Variables		Percentage of Respondents User of Medicinal Plants (*n* = 113)
Gender	Male	5.3
	Female	94.7
Education status	No schooling	24.8
	Primary	62.8
	Secondary	11.5
	Post-secondary	0.9
Age	40–50 years	31.9
	51–60 years	35.4
	61–70 years	22.1
	71 and more years	10.6
Household income/month (U$)	≤300.00	34.5
	300.00–600.00	58.4
	≥600.00	7.1
Number of Plants used	1–3	77.9
	4–6	16.8
	7–9	5.3
Obtaining Plant site	Neighbors/friends/family	53.0
	Drugstore/Supermarket	8.5
	Raizeiro *	38.5

* Raizeiro are generally people with little formal education, who through the knowledge transmitted by their parents or other people with empirical knowledge, use plants for disease treatment.

**Table 2:** Ethnomedicinal uses of plant species from Maceió (Alagoas-Brazil).

*Plant Species* Popular Name	Family	Plant Part Used	Method of Preparation	Use Mode	Therapeutic Uses	UV
***Justicia pectoralis* Jacq.** Anador	Acanthaceae	Leaves	Decoction	Oral	Gastrointestinal diseases and pain	0.04
***Sambucus nigra* L.** Sabugueiro	Adoxaceae	Leaves, flower	Infusion, decoction, caramelized fluid	Oral	Respiratory diseases and fever	0.05
***Allium sativum* L.** Alho	Alliaceae	Bulbs	Infusion, decoction, macerated/water	Oral	Respiratory diseases and expectorant	0.40
***Aloe vera* L.** Babosa	Aloeaceae	Leaves	Cataplasm, infusion, decoction	Topic, oral	Healing	0.02
***Schinus terebinthifolius* Raddi** Aroeira	Anacardiaceae	Leaves	Decoction, juice, macerated/alcohol	Oral, washer	Uterus inflammation, colic, sore throat and healing	0.06
***Anonna muricate* L.** Graviola	Annonaceae	Leaves	Decoction, infusion	Oral	Gastrointestinal and cardiovascular diseases and pain	0.02
***Annona squamosa* L.** Pinha	Annonaceae	Leaves	Decoction	Oral	Pain and dysentery	0.02
***Pimpinella anisum* L.** Erva doce	Apiaceae	Fruits	Infusion, decoction	Oral	Calmative, bloated belly, colic and gastrointestinal diseases	0.28
***Baccharis trimera* Less.** Carqueja	Asteraceae	Leaves	Infusion, decoction	Oral, topic	Cardiovascular and gastrointestinal diseases and diabetes	0.03
***Cynara scolymus* L.** Alcachofra	Asteraceae	Leaves	Infusion, decoction	Oral	Gastrointestinal diseases and slimming	0.03
***Matricaria recutita* L.** Camomila	Asteraceae	Flower	Infusion, decoction	Oral	Calmative and digestive	0.07
***Pluchea sagittalis* Lam.** Quitoco	Asteraceae	Flower, Leaves	Decoction	Oral	Inflammation and pain	0.02
***Tarenaya spinosa*** Muçambê	Capparaceae	Flower	Infusion, decoction	Oral	Respiratory diseases and allergy	0.03
***Chenopodium ambrosioides*****L.** Mastruz	Chenopodiacea	Leaves	Caramelized fluid, juice, decoction	Oral, topic	Pain, headache, respiratory diseases, flu, bloated belly, intestinal infection and colic	0.04
***Vismia guianensis* Aubl.** Lacre	Clusiaceae	Stem-root	Macerated/water, bottled	Oral	Cancer	*
***Momordica charantia*****L.** Melãozinho	Cucurbitaceae	Leaves, flower	Decoction, macerated/water	Topic	Haemorrhoids and scabies	*
***Abarema cochliacarpos*****(Gomes) Barneby & Grimes** Barbatimão	Fabaceae	Stem-bark, leaves	Decoction, macerated/alcohol	Oral, washes	Pain, inflammation and healing	0.05
***Cajanus cajan*****L.** Feijão andú	Fabaceae	Leaves	Decoction	Oral	Cardiovascular disease	0.02
***Senna alexandrina*****Mill.** Sene	Fabaceae	Leaves	Infusion, decoction	Oral	Indigestion, constipation and gastrointestinal diseases	0.04
***Endopleura uchi*****Huber** Uchi-amarelo	Humiriaceae	Root	Decoction	Oral	Healing	*
***Coleus amboinicus*****Lour.** Hortelã da folha grande	Lamiaceae	Leaves	Infusion, decoction, caramelized fluid	Oral	Respiratory diseases, flu, cough, pain and dysentery	0.07
***Hyptis pectinate*****(L.) Poit** Sambacaitá	Lamiaceae	Leaves	Infusion, decoction, juice	Oral, washes	Gastrointestinal and respiratory diseases, healing, anti-inflammatory and menstrual colic	0.17
***Mentha piperita*****L.** Hortelã da folha miúda	Lamiaceae	Leaves, Flowers	Infusion, decoction, caramelized fluid	Oral	Menstrual colic, healing, headache, fever, pain, respiratory, cardiovascular and gastrointestinal diseases	0.30
***Ocimum Basilicum*****L.** Mangericão	Lamiaceae	Leaves	Decoction	Oral	Respiratory disease	0.05
***Ocimum champechianum*****Mill.** Alfavaca	Lamiaceae	Leaves	Decoction, fluid caramelized	Oral, washes	Flu, cough, dysentery, pain, gastrointestinal and cardiovascular diseases	0.07
***Plectranthus barbatus*****Andr.** Boldo	Lamiaceae	Leaves	Infusion, decoction	Oral	Gastrointestinal diseases and pain	0.71
***Plectranthus ornatus*****Codd.** Boldo selvagem	Lamiaceae	Leaves	Decoction	Oral	Gastrointestinal disease	0.05
***Rosmarinus officinalis*****L.** Alecrim	Lamiaceae	Leaves	Infusion, decoction	Oral	Pain, menstrual colic and inflammation	0.02
***Cinnamomum verum*****J. Presl** Canela	Laureaceae	Stem-bark, leaves	Infusion, decoction	Oral	Nausea, healing, diabetes, pain and gastrointestinal disease	0.14
***Persea Americana*****Mill.** Abacate	Laureaceae	Leaves	Decoction	Oral	Diuretic and kidney disease	0.04
***Punica granatum*****L.** Romã	Lythracae	Bark-fruit, seeds	Decoction, fresh fruit	Oral	Respiratory disease	0.13
***Eucalyptus tereticornis*****Sm.** Eucalipto	Myrtaceae	Leaves, fruits	Infusion, decoction	Oral	Flu, respiratory diseases and pain	0.01
***Eugenia uniflora*****L.** Pitangueira	Myrtaceae	Leaves	Decoction	Oral	Healing	0.05
***Psidium guajava*****L.** Goiabeira	Myrtaceae	Leaves	Decoction	Oral	Gastrointestinal disease	0.01
***Averrhoa carambola*****L.** Carambola	Oxalidaceae	Fruits	Juice	Oral	Dysentery	0.02
***Passiflora edulis*****Sims.** Maracujá	Passifloraceae	Leaves, fruits	Decoction, juice	Oral	Respiratory and cardiovascular diseases, calmative and insomnia	0.11
***Petivera Alliaceae*****L.** Tipi	Phytolaccacea	Leaves	Infusion, decoction	Oral	Gastrointestinal diseases, pain and fever	0.04
***Phyllanthus Niruri*****L.** Quebra-pedra	Phyllanthaceae	Whole plant, root	Infusion, decoction	Oral	Kidney disease	0.11
***Cymbopogon citratus*****DC.** Capim-santo	Poaceae	Leaves	Infusion, decoction macerated/water	Oral	Pain, calmative, flu, insomnia, gastrointestinal diseases and anti-inflammatory	0.62
***Uncaria tomentosa*****Willd.** Unha-de-gato	Rubiaceae	Stem-root	Infusion, decoction	Oral	Healing and urinary infection	0.02
***Ruta graveoles*****L.** Arruda	Rutaceae	Leaves	Decoction, macerated/water	Oral, washer	Pain	0.02
***Citrus sinensis*****L.** Laranjeira	Rutaceae	Leaves	Decoction	Oral	Calmative	0.02
***Camellia sinensis*****L.** Chá verde	Theaceae	Leaves	Decoction	Oral	Gastrointestinal diseases	0.02
***Tunera subulate*****Sm.** Garrida	Turneraceae	Leaves	Decoction	Oral	Gastrointestinal diseases	0.03
***Cecropia hololeuca*****Miq.** Embaúba-branca	Urticaceae	Flower, fruits	Macerated/water	Oral	Diabetes	0.03
***Lippia alba*****Mill.** Erva-cidreira	Verbenaceae	Leaves	Infusion, decoction, macerated/water	Oral	Calmative, pain, insomnia, headache, gastrointestinal and kidney diseases, expectorant and malaise	0.81
***Zingiber officinale*****Roscoe** Gengibre	Zingiberaceae	Root, leaves	Decoction	Oral	Pain, respiratory and gastrointestinal diseases	0.04
***Alpinia zerumbet*****Pers.** Colônia	Zingiberaceae	Leaves, flower	Infusion, decoction	Oral	Agony, cardiovascular diseases and calmative	0.04

*n* = 113 formulary of interview. * This information was not considered for analysis by have been obtained from a single informant.

**Table 3:** Informant consensus factor (ICF) values of category of ailments.

No.	Category	Number of Use (Nur)	All Use Citations (%)	Number of Taxa (Nt)	ICF
1	Nervous system disorders	65	22.0	8	0.89
2	Fever	17	5.7	4	0.81
3	Gastrointestinal disorders	92	31.1	24	0.75
4	Urogenital disorders	8	2.7	3	0.70
5	Pain	54	18.3	20	0.64
6	Healing and inflammation	25	8.5	13	0.64
7	Respiratory diseases	21	7.1	15	0.63
8	Diabetes	4	1.3	3	0.33
9	Cardiovascular diseases	9	3.0	8	0.13
10	Cancer	1	0.3	1	0.00
